# The African Women's Protocol: Bringing Attention to Reproductive Rights and the MDGs

**DOI:** 10.1371/journal.pmed.1000429

**Published:** 2011-04-05

**Authors:** Liesl Gerntholtz, Andrew Gibbs, Samantha Willan

**Affiliations:** 1 Human Rights Watch, New York, United States of America; 2 Health Economics and HIV/AIDS Research Division (HEARD), University of KwaZulu-Natal, Durban, South Africa

## Abstract

Andrew Gibbs and colleagues discuss the African Women's Protocol, a framework for ensuring reproductive rights are supported throughout the continent and for supporting interventions to improve women's reproductive health, including the MDGs.

**Summary Points**

- Despite overall progress on the Millennium Development Goals (MDGs), Goal 3 (promote gender equality), Goal 5 (reduce maternal mortality), and Goal 6 (combat HIV/AIDS, malaria, and other diseases) significantly lag behind other goals, with women in Africa bearing the burden of this failure.
- Underlying this lack of progress is the failure to protect and promote women's reproductive rights.
- The Protocol to the African Charter on Human and Peoples' Rights on the Rights of Women in Africa (or, the African Women's Protocol) provides a strong, African framework for women's reproductive rights that goes beyond other binding international treaties in supporting and promoting reproductive rights.
- Only 29 out of 52 countries in Africa have signed and ratified the African Women's Protocol thus far, and there remain significant barriers to translating the Protocol into national legislation and implementing its provisions.
- If fully implemented and integrated into national legislation, the African Women's Protocol offers a significant tool to support women's reproductive rights in Africa, thereby supporting the attainment of MDGs 3, 5, and 6.

## Lack of Progress on All Millennium Development Goals for Women

The international community recently reviewed 10 years of progress towards the Millennium Development Goals (MDGs). The outcome document of the High Level Plenary Meeting of the General Assembly, adopted by a consensus of the General Assembly of the United Nations on 22 September 2010, recognised the significant steps made towards achieving many of the goals, but also emphasised the uneven progress and that more must be done to ensure that the MDGs will be met in 2015 [1].

Significantly, the fifth goal—to improve maternal health—has made the least progress, with 350,000 women still dying annually of pregnancy-related causes [2]; the MDG outcome document expresses “grave concern over the slow progress being made on reducing maternal mortality and improving maternal and reproductive health” [1]. While greater progress has been made with regard to MDG 6, to combat HIV/AIDS, malaria, and other diseases, and MDG 3, to promote gender equality, progress in these two MDGs remains limited.

The lack of progress across MDGs 3, 5, and 6 is linked; failure to progress in any of these three MDGs undermines progress in the other two. Despite global progress in reducing maternal mortality, the impact of HIV/AIDS has slowed reductions in maternal mortality and in some countries increased maternal mortality [2]. One estimate suggests HIV contributed an additional 64,100 maternal deaths globally in 2008 [3]. Furthermore, both AIDS-related morbidity and mortality and maternal mortality undermine women's ability to realise their equality by excluding women from education and employment. While all women are ill-served by this lack of progress, women in Africa, who are especially vulnerable, will bear a disproportionate burden of these failures [2],[4],[5].

## The Forgotten Millennium Goal: Improving Women's Reproductive Rights

Underlying the failure to meaningfully progress towards achieving MDGs 3, 5, and 6, particularly in Africa, is the failure to protect and promote women's human rights, including their reproductive rights. The United Nations Population Fund [6] outlines the three components of reproductive rights: the right to control sexual and reproductive lives, the right to non-discrimination, and the right to reproductive health care. This creates a framework that supports women's rights to insist and engage in safer sex (including the right to be free from unwanted sex) and to access comprehensive and accurate information on HIV/AIDS and family planning and comprehensive reproductive health care, which includes termination of pregnancy and post-abortion care. Furthermore, reproductive rights are critical in ensuring that women can control their fertility and in supporting their participation in social, economic, and political life.

While earlier international treaties such as the Convention on the Elimination of Discrimination of Discrimination Against Women (CEDAW) [7] and the International Convention on Economic, Social and Cultural Rights [8] supported aspects of reproductive rights without explicitly referring to them, it was in Cairo in 1994 at the International Conference on Population and Development (ICPD) [9], and again in 1995 at the Fourth World Conference on Women in Beijing [10], that reproductive rights and their relationship to women's rights and development broadly was firmly established. The ICPD Programme of Action remains a foundational vision for women's reproductive health, committing the 179 participating nations to the achievement of universal and equal access to reproductive health by 2015. The definition of reproductive health in the ICPD Programme of Action was broad and inclusive and included family planning services and counselling, comprehensive sexuality education, and maternal and child health services. The Beijing conference further amplified links between women's ability to participate fully in all spheres of life and their reproductive rights, and reiterated the importance of achieving the goal of universal access.

More recent international treaties, such as the Convention on the Rights of People with Disabilities [11] and the Convention on the Rights of the Child [12], have built on and reaffirmed a global commitment to achieving reproductive rights. In 2010, the UN Human Rights Council passed a second resolution on maternal mortality that reaffirmed the need to protect women's human rights as part of a comprehensive strategy to address maternal mortality and morbidity [13].

Despite recognition of the key role that reproductive rights plays in advancing women's health and empowerment, the MDGs originally failed to include a specific goal on access to reproductive health care or reproductive rights. Partly in response to lobbying by women's rights activists, access to reproductive health was added as one of the targets in MDG 5 at the 5-year review meeting of the MDGs in 2005. Indeed, target 5B reaffirms the commitment made at the ICPD to achieve universal access to reproductive health by 2015. That the importance of women's reproductive rights to women's health, autonomy, and ability to participate fully in society has been recognised for decades makes the absence of significant attention to these rights in the MDGs even more deplorable.

## An Enabling Environment for Women's Reproductive Rights in Africa

Currently, there are a range of issues that undermine women's reproductive rights in Africa. Specific issues include:

- Criminalisation of HIV transmission [14],[15]. At present, in Africa and globally, a number of countries have either passed legislation, or are considering legislation, that criminalises the transmission of HIV. Criminalisation does little to reduce HIV transmission [14],[15] and disproportionately affects women, who are often unable to decide how and when sex occurs [16]. Women may also be less willing to disclose their HIV status to sexual partners for fear of violence and abandonment. Criminalisation also reduces access to reproductive health services, especially for vulnerable groups of women such as sex workers and adolescents;
- Anti-abortion legislation [17]. Such legislation limits women's ability to determine whether and when to have children;
- High levels of violence against women, often in contexts of weak or limited legislative frameworks to support women's rights [18],[19]. Violence against women limits their autonomy and ability to make decisions about their body and sex. Furthermore, violence places women at greater risk of acquiring HIV, and may make them weary of accessing reproductive health services and HIV testing. The failure to criminalise marital rape in many African counties has increased the risk of HIV transmission for married women and undermines their access to post-exposure prophylaxis;
- Limited rights to comprehensive sexuality education and access to male and female condoms, particularly for young people [20]. The failure to provide comprehensive sexuality education and access to male and female condoms undermines women's abilities to make fully informed reproductive choices and act on these decisions; and
- Failure in many national policies to realise the reproductive rights of women (and men) living with HIV [21], seen most explicitly in the emergence of coerced or forced sterilisation of women living with HIV [22]. Without such a right, women living with HIV/AIDS have a reduced ability to make reproductive decisions.

The many barriers to the promotion and protection of women's reproductive rights in Africa undermines women's ability to take control of their sexual health, fertility, autonomy, and participation in social and economic life. Technical, discrete interventions to promote women's health, tackle HIV/AIDS, and reduce maternal mortality are unlikely to work if wider laws and policies continue to undermine women's reproductive rights.

## The African Women's Protocol

The Protocol to the African Charter on Human and Peoples' Rights on the Rights of Women in Africa [23] (the African Women's Protocol), which was adopted by the African Union in 2003 and became legally binding for countries that had signed and ratified the protocol on 25 November 2005, corrects the weaknesses in the African Charter on Human and People's Rights (African Charter) [24] with respect to women's rights. While the African Charter provides an important human rights framework, including reinforcing the right to life, liberty, security and freedom from discrimination, it is silent about women's rights in general and reproductive rights specifically [25]. The protocol promotes women's rights and equality broadly, including in marriage and divorce, land tenure, inheritance rights, and in relation to “traditional” practices. The protocol contains specific protections for older women, disabled women, and women in distress, but fails to include similar provisions for girls and young women.

The African Women's Protocol emerged through extensive lobbying of government by women's rights organisations across Africa and around the world. Since 1995, African women's rights activists have recognised the limitation of the African Charter and called on the Organisation of African Unity (OAU) to address the rights of women with a specific instrument. In response, the OAU mandated the African Commission on Human and People's Rights to develop a protocol. A draft was circulated to non-governmental organisations for comment in 1997 and the Commission later endorsed the appointment of a special rapporteur on women's rights to finalise the protocol. Following consultations with civil society, the text was revised and adopted in 2005.

The African Women's Protocol is particularly strong on women's reproductive rights, and is a tool for ensuring universal access to reproductive health and the creation of an enabling environment. It goes beyond other binding treaties, such as CEDAW, in outlining reproductive rights [25]. It contains the first references to HIV/AIDS in an international treaty, and the first expression of a right to abortion, albeit limited to where a pregnancy is the result of sexual assault, rape, or where it endangers a woman's mental or physical health. It specifically recognises marital rape as a form of gender-based violence. Moreover, the protocol “identifies protection from HIV and AIDS as a key component of women's sexual and reproductive rights” [25].

Articles 14(1&2) of the African Women's Protocol set out three major components of women's reproductive health rights:

1. Reproductive and sexual decision making, including the number and spacing of children, contraceptive choice, and the right to self-protection from HIV;
2. Access to information about HIV/AIDS and reproductive health; and
3. Access to reproductive health services, including antenatal services and abortion-related services.

Unfortunately, the vision offered by the African Women's Protocol is still to be realised across Africa. Only 29 out of 52 of the African Union countries have currently signed and ratified the protocol (see Table 1). While ratification is a significant step, it is, however, only the first step in utilising the African Women's Protocol to realise women's reproductive rights. Countries must domesticate the protocol—that is, translate the protocol into national (domestic) legislation. This would require a comprehensive legal review of the provisions of the protocol in relation to current legislation. Countries' failures to sign, ratify, and domesticate the protocol are major barriers in utilising this legislation. Countries frequently raise concerns about the cost of reviewing legislation and implementing reproductive rights. A further barrier is continued tension between human rights and customary laws.

**Table 1:** African Union countries and the signing and ratification of the African Women's Protocol.

Not Signed or Ratified	Signed Only^a^	Ratified
Botswana	Algeria	Angola
Egypt	Burundi	Benin
Eritrea	Cameroon	Burkino Faso
Tunisia	Central African Republic	Cape Verde
	Chad	Comores
	Cote d'Ivoire	Dijbouti
	Equatorial Guinea	Democratic Republic of Congo
	Ethiopia	Gambia
	Gabon	Ghana
	Guinea	Guinea-Bissau
	Madagascar	Kenya
	Mauritius	Libya
	Niger	Lesotho
	Sahrawi Arab Democratic Republic	Liberia
	Sierra Leone	Mali
	Somalia	Malawi
	Sao Tome & Principe	Mozambique
	Sudan	Mauritania
	Swaziland	Namibia
		Nigeria
		Rwanda
		South Africa
		Senegal
		Seychelles
		Tanzania
		Togo
		Uganda
		Zambia
		Zimbabwe

a The signature of a treaty is evidence of the state's intention to ratify the instrument at some time in the future, but is not legally binding for a state.

Finally, legislative change is meaningless if it is not implemented, monitored, and enforced to ensure real change for women in Africa [26]. Strengthening and ensuring the utilisation of accountability mechanisms contained in the African Women's Protocol and guaranteeing that they are replicated at a national level is crucial. Upon ratification, countries are bound to report on steps taken to fulfil their obligations when they report more widely on the African Charter. Yet reporting by governments lacks transparency and is limited.

Learning from CEDAW, civil society has a crucial role to play in ensuring accountability and implementation, including production of shadow reports and active lobbying of governments to encourage further implementation. Civil society may, however, remain constrained without significant resources being allocated to this specific role.

Through the ratification, domestication, and transparent reporting on the African Women's Protocol, a framework can be implemented in Africa that creates contexts that support women's reproductive rights. In so doing, a significant step can be made towards rolling back HIV and maternal mortality and thereby supporting the attainment of MDGs 3, 5, and 6.

## Abbreviations

CEDAW: Convention on the Elimination of Discrimination Against Women
ICPD: International Conference on Population and Development
MDG: Millennium Development Goal
OAU: Organisation of African Unity
